# Sex- and age-specific percentiles of body composition indices for Chinese adults using dual-energy X-ray absorptiometry

**DOI:** 10.1007/s00394-016-1279-9

**Published:** 2016-07-29

**Authors:** Zeyu Xiao, Bin Guo, Jian Gong, Yongjin Tang, Jingjie Shang, Yong Cheng, Hao Xu

**Affiliations:** Department of Nuclear Medicine, the First Affiliated Hospital, Jinan University, Guangzhou, China

**Keywords:** Body composition, Percentile curves, Body fat distribution, Nutritional assessment, Dual-energy X-ray absorptiometry

## Abstract

**Purpose:**

The aims of the study were to develop sex- and age-specific percentiles for lean mass index (LMI), appendicular LMI (aLMI), fat mass index (FMI), and body fat distribution indices in Chinese adults using dual-energy X-ray absorptiometry (DXA), and to compare those indices with those of other ethnicities using the US NHANES data.

**Methods:**

Whole-body and regional lean mass and fat mass (FM) were measured using DXA in 5688 healthy males (*n* = 1693) and females (*n* = 3995) aged 20–90 years. Body fat distribution indices were expressed as % fat trunk/% fat legs, trunk/appendicular FM ratio (FMR), and android/gynoid FMR. Percentile curves of LMI, aLMI, FMI, and body fat distribution indices were obtained by the Lambda–Mu–Sigma method.

**Results:**

The aLMI and LMI were negatively associated with age, decreasing from the fifth decade for males, but were not associated with age in females. Females had more total FM than males, whereas males had greater central adiposity (% fat trunk/% fat legs ratio, trunk/appendicular FMR, and android/gynoid FMR) than females. Moreover, FMI and body fat distribution indices consistently increased with age in both sexes, especially in women. In comparison with white, black, and Mexican populations in the USA, Chinese adults had lower total FM, but had greater central adiposity (% fat trunk/% fat legs ratio and trunk/appendicular FMR). Additionally, older white and Mexican populations showed greater decreases for aLMI and LMI than their Chinese counterparts.

**Conclusions:**

We present the sex- and age-specific percentiles for aLMI, LMI, FMI, and body fat distribution indices by DXA in Chinese adults, which may refine the individual assessment of the nutritional status of Chinese adults.

## Introduction

Assessment of nutritional status provides a useful predictor of health risk and an opportunity to monitor the effects of nutrition-related disease progression and nutritional intervention in public health and clinical nutrition [1, 2]. BMI has been widely used in epidemiological studies and clinical practice to provide a quick assessment of nutritional status [3]. However, BMI represents the sum of total body composition. The failure to differentiate between lean mass (LM) and fat mass (FM) limits the usefulness of BMI, which may lead to significant misclassification of nutrition status when applied to individuals [4]. To overcome the limitations of BMI and to obtain more phenotypic details, body composition analysis (including LM and FM) is necessary. Measurements of LM and FM in absolute values fail to allow appropriate comparisons among subjects of different sizes. Therefore, some studies have proposed the use of lean mass index (LMI) and fat mass index (FMI) normalized to height as superior measures of nutritional status [5]. In previous studies, FMI was widely used to screen individuals with obesity-risk diseases [6, 7], and LMI (especially in limbs) has been applied as an important index for diagnosis of sarcopenia [8, 9].

Dual-energy X-ray absorptiometry (DXA) is an accurate and reliable method for assessing body composition, and is capable of separating body mass into LM and FM [10]. Moreover, DXA has the ability to measure on both a regional and whole-body basis with the advantages of low cost, low radiation dose, and high precision [10]. Recently, reference data based on DXA were published for LMI and FMI (total body and regional) in different ethnic populations [11–13]. The most advanced and comprehensive reference values for body composition are age-, sex-, and ethnicity-specific and have been published as percentiles derived from the US NHANES data [11, 12]. We previously described reference data of body composition in healthy Chinese children and adolescents aged 5–19 years using DXA [14]. To the best of our knowledge, there are currently no DXA-based sex- and age-specific data for LMI and FMI in a population of Chinese adults.

Previous studies have shown that Asian populations experience a higher risk of metabolic and cardiovascular diseases at lower levels of BMI than other ethnic populations [15, 16]. This paradoxical finding conveyed the necessity for additional adiposity measures to supplement BMI in assessing health risk, such as measures of body fat distribution that are more strongly associated with risk factors for obesity-related diseases than total fat mass [17]. Moreover, ethnic differences in body fat distribution exist in adults [11, 12, 18]. Thus, ethnicity-specific percentile curves of body fat distribution indices would further assist in the assessment of nutritional status in adults.

Therefore, the primary aim of the current study was to develop sex- and age-specific percentiles for LMI, FMI, and body fat distribution indices in a population of Chinese adults aged 20–90 years using DXA. A secondary aim was to compare the body composition indices of the Chinese population with those of other ethnic populations.

## Methods

### Study population

The participants were recruited from a body composition and osteoporosis study at the First Affiliated Hospital of Jinan University (Guangzhou, China) from 2004 to 2012. The present study included healthy Chinese men and women from 20 to 90 years of age who had a BMI of 16–30 kg/m^2^. Subjects were included in the study if they were functionally independent Chinese individuals over 20 years of age, who were in apparent good health. Subjects were excluded if they met any of the following criteria: (1) a history of fracture; (2) medication known to affect the musculoskeletal system (e.g., anti-osteoporotic drugs, androgens or anti-androgen drugs, corticosteroids); (3) chronic disease known to affect bone metabolism (e.g., hyperthyroidism, hyperparathyroidism, rheumatoid arthritis, chronic renal insufficiency); (4) metal implants (e.g., pacemakers, joint replacement device); or (5) inability to determine the menstruation state or experiencing non-natural menopause (natural menopause was designated if there was a complete natural cessation of menses for more than 12 months). Ultimately, 1693 men and 3995 women were included in our study. All subjects provided written informed consent to participate in the study, which was approved by the Ethics Committee of the First Affiliated Hospital, Jinan University.

### Anthropometry and body composition measurement

A research physician obtained information on medical history and medication use in a personal interview. Height and body weight were obtained based on standard methods; height was measured without shoes to the nearest 0.1 cm, and weight with only light clothing to the nearest 0.1 kg. BMI was calculated as body weight divided by height squared (kg/m^2^). Total and regional LM and FM were obtained through whole-body DXA scans (Lunar Prodigy, GE Healthcare, Madison, WI, USA), and data were analyzed using software version 10.0 provided by the manufacturer. Android and gynoid regions were automatically obtained using the software provided by the manufacturer. The appendicular region is defined as including both the left and right arms and legs area. From these measurements, the following derivative values were calculated: FMI (total fat mass/height^2^), LMI (total lean mass/height^2^), aLMI (appendicular lean mass/height^2^), and % BF (percentage of body fat mass = total body fat mass/weight × 100 %). Indices of body fat distribution were included in the analysis as previously described: % fat trunk/% fat legs, trunk/appendicular FM ratio (FMR), and android/gynoid (A/G) FMR. Daily quality assurance scans were performed by scanning the spine phantom according to the manufacturer’s instructions. All DXA measurements were taken by the same trained technologist throughout the study. The precision error (% CV) was less than 2 % for total LM and total FM and less than 3 % for regional (trunk, appendicular, android, gynoid) LM and FM, as determined by duplicate scans with repositioning between each measurement in 30 volunteer subjects.

### Statistical analysis

The values of continuous variables are presented as the mean ± SD. Unpaired-sample *t* tests were conducted to evaluate the significance of the mean difference between males and females. Age- and sex-specific percentile curves for aLMI, LMI, FMI, % BF and indices of body fat distribution were generated using the Lambda–Mu–Sigma method (LMS-chartmaker version Pro 2.3, Medical Research Council, UK). Each percentile (changing distribution) is summarized by three curves representing the skewness (L), the median (M), and the coefficient of variation (S) as these change with the independent variable age [19]. The *Z*-scores can be calculated by the following equation:

$$ Z = \frac{{{\text{M }}\left( {\left( {\frac{X}{M}} \right)^{L} - 1} \right)}}{Ls}, $$

where *X* is the body composition measure of interest.

The 50th percentile curves for aLMI, LMI, FMI, and body fat distribution indices (including % fat trunk/% fat legs ratio and trunk/appendicular FMR) of Chinese adults were compared with those of white, black, and Mexican populations in the US NHANES reference data using the same GE-Lunar DXA scanner [12]. The comparisons did not include the A/G FMR because the NHANES reference data did not provide these values. All tests were two-tailed, and a *p* value of less than 0.05 was considered statistically significant.

## Results

### Characteristics of subjects

Details of the subject characteristics are shown in Table 1. Males and females differed significantly in weight and height as well as in total and regional LM and FM. Males were heavier, were taller, and had higher LM, whereas FM was greater in females.

**Table 1:** Characteristics of study subjects

	Males (*n* = 1693)	Females (*n* = 3995)	Total (*n* = 5668)
	Mean ± SD	Mean ± SD	Mean ± SD
Age (years)	52.8 ± 17.5^c^	54.0 ± 15.9	53.6 ± 16.4
Weight (kg)	63.5 ± 10.3^c^	53.8 ± 8.1	56.7 ± 9.9
Height, m	1.68 ± 0.06^c^	1.57 ± 0.05	1.60 ± 0.08
BMI (kg/m^2^)	22.5 ± 3.2^b^	21.9 ± 3.0	22.1 ± 3.1
Body composition measures (kg)			
Total LM	46.72 ± 6.00^c^	33.93 ± 3.78	37.73 ± 7.32
Total FM	14.27 ± 6.47^c^	17.86 ± 5.65	16.80 ± 6.13
Total BMC	2.527 ± 0.433^c^	1.978 ± 0.384	2.142 ± 0.472
Trunk LM	22.39 ± 2.64^c^	16.76 ± 2.04	18.44 ± 3.41
Trunk FM	8.62 ± 4.30^c^	9.76 ± 3.48	9.42 ± 3.78
Appendicular LM	20.52 ± 3.11^c^	14.12 ± 1.97	16.03 ± 3.76
Appendicular FM	5.07 ± 2.19^b^	7.39 ± 2.35	6.70 ± 2.54
Body composition index (kg/m^2^)			
Total LMI	16.6 ± 1.6^c^	13.8 ± 1.3	14.6 ± 1.9
Total FMI	5.1 ± 2.2^a^	7.3 ± 2.2	6.6 ± 2.5
Appendicular LMI	7.3 ± 0.9^c^	5.7 ± 0.7	6.2 ± 1.0
Body fat distribution			
% BF	21.62 ± 7.62^c^	32.55 ± 6.49	29.30 ± 8.48
% Fat trunk/% fat legs ratio	1.33 ± 0.30^c^	1.08 ± 0.19	1.16 ± 0.25
Trunk/appendicular FMR	1.66 ± 0.43^c^	1.33 ± 0.34	1.43 ± 0.39
A/G FMR	0.63 ± 0.19^c^	0.51 ± 0.15	0.54 ± 0.17
BMI status (%)			
BMI < 18.5 kg/m^2^	12.3	13.8	13.4
BMI ≥ 25 kg/m^2^	23.3	16.2	18.4

*BMI* body mass index, *LM* lean mass, *FM* fat mass, *BMC* bone mineral content, *LMI* lean mass index, *FMI* fat mass index, *% BF* percentage of body fat mass, *FMR* fat mass ratio, *A/G* android/gynoid

^a^ *P* ≥ 0.05; ^b^ *P* < 0.01; ^c^ *P* < 0.001. Compared with females (unpaired-sample *t* tests)

### Percentiles for LMI, FMI, aLMI, and % BF

**Fig. 1:**
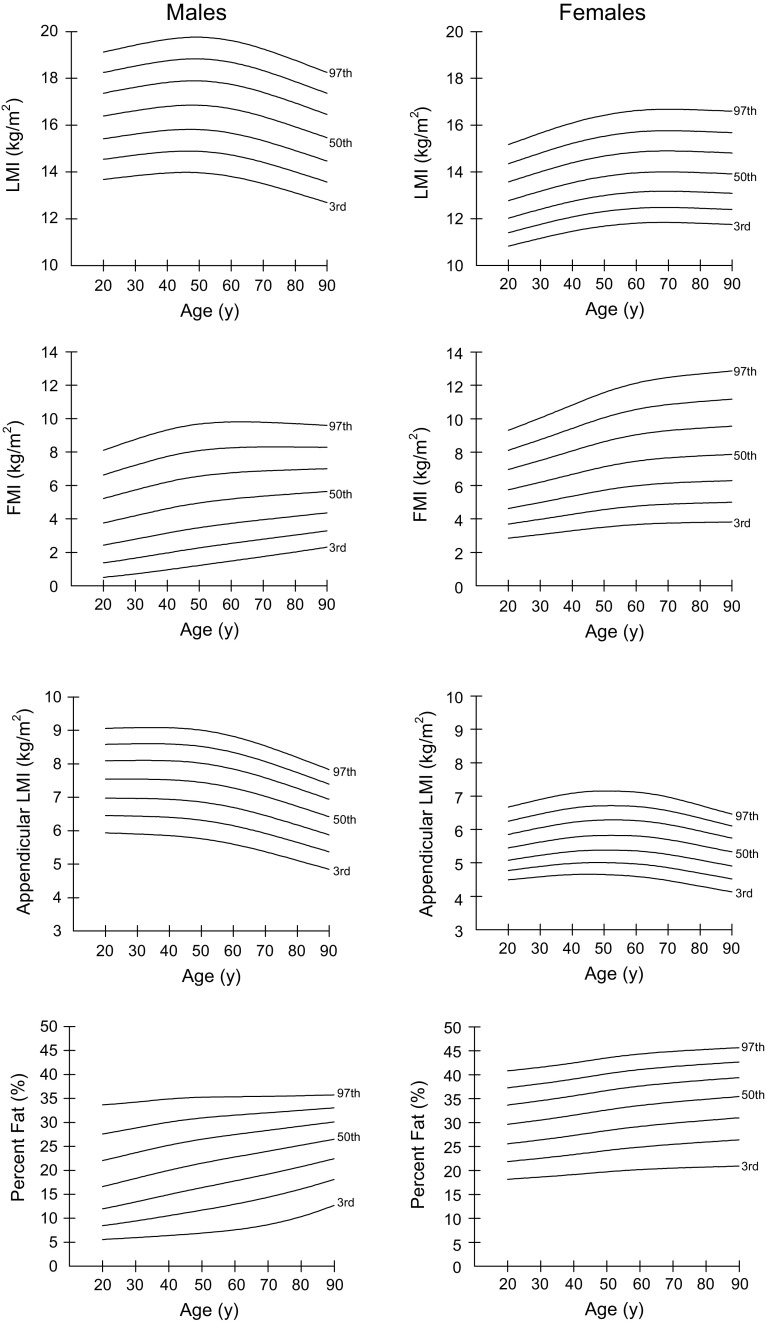
Percentile curves (the 3rd, 10th, 25th, 50th, 75th, 90th, and 97th) for LMI, FMI, appendicular LMI, and % body fat in Chinese males and females aged 20–90 years. (*LMI* lean mass index, *FMI* fat mass index, *% BF* percentage of body fat mass)

Percentiles (3rd, 10th, 25th, 50th, 75th, 90th, and 97th) for LMI, FMI, aLMI, and % BF for males and females aged 20–90 years are shown in Tables 2, 3, 4 and 5, and percentile curves are given in Fig. 1. The differences between sexes were seen in percentile curves for LMI, FMI, aLMI, and % BF. The aLMI and LMI were consistently higher in males of all age groups than females, whereas the FMI and % BF were consistently higher in females. The aLMI (*r* = −0.123, *P* < 0.001) and LMI (*r* = −0.292, *P* < 0.001) were negatively associated with age and started to decrease from the fifth decade in men, whereas in women the aLMI and LMI had no correlation with age, although a slow decrease from the sixth decade was seen for aLMI. The FMI and % BF were positively related to age and showed a steady increase with age in both sexes.

**Table 2:** Sex- and age-specific percentiles for lean mass index among Chinese adults aged 20–90 years

Age (years)	Males									Females								
			M									M						
	L	S	3rd	10th	25th	50th	75th	90th	97th	L	S	3rd	10th	25th	50th	75th	90th	97th
20	0.957	0.088	13.68	14.54	15.42	16.39	17.37	18.25	19.13	−0.243	0.090	10.83	11.41	12.03	12.77	13.58	14.35	15.17
25	0.957	0.089	13.76	14.64	15.52	16.51	17.50	18.40	19.28	-0.243	0.090	11.00	11.58	12.22	12.98	13.79	14.58	15.42
30	0.957	0.089	13.84	14.73	15.62	16.62	17.63	18.53	19.43	−0.243	0.090	11.16	11.76	12.41	13.18	14.01	14.81	15.66
35	0.957	0.090	13.91	14.80	15.71	16.73	17.74	18.66	19.56	−0.243	0.090	11.32	11.93	12.58	13.37	14.21	15.03	15.89
40	0.957	0.090	13.96	14.86	15.78	16.81	17.83	18.76	19.67	−0.243	0.090	11.46	12.08	12.75	13.54	14.40	15.22	16.10
45	0.957	0.091	13.98	14.90	15.82	16.85	17.89	18.82	19.75	−0.243	0.090	11.59	12.21	12.88	13.69	14.55	15.39	16.28
50	0.957	0.091	13.97	14.89	15.82	16.86	17.90	18.84	19.76	−0.243	0.091	11.68	12.31	12.99	13.81	14.68	15.53	16.43
55	0.957	0.092	13.91	14.83	15.77	16.81	17.85	18.79	19.72	−0.243	0.091	11.76	12.39	13.08	13.90	14.78	15.64	16.54
60	0.957	0.092	13.81	14.73	15.66	16.70	17.75	18.69	19.62	−0.243	0.091	11.81	12.45	13.14	13.96	14.85	15.71	16.62
65	0.957	0.093	13.67	14.59	15.52	16.56	17.60	18.53	19.46	−0.243	0.091	11.84	12.48	13.17	14.00	14.89	15.75	16.67
70	0.957	0.094	13.50	14.41	15.34	16.37	17.40	18.34	19.26	−0.243	0.091	11.84	12.48	13.18	14.00	14.90	15.77	16.68
75	0.957	0.094	13.31	14.22	15.14	16.16	17.18	18.11	19.03	−0.243	0.091	11.83	12.47	13.16	13.99	14.89	15.76	16.67
80	0.957	0.095	13.11	14.01	14.92	15.93	16.95	17.87	18.78	−0.243	0.091	11.81	12.45	13.14	13.97	14.87	15.73	16.65
85	0.957	0.095	12.90	13.79	14.69	15.70	16.71	17.61	18.51	−0.243	0.092	11.78	12.42	13.12	13.94	14.84	15.71	16.63
90	0.957	0.096	12.70	13.58	14.47	15.47	16.46	17.36	18.26	−0.243	0.092	11.75	12.40	13.09	13.92	14.81	15.68	16.60

L (lambda), skewness; M (mu), median; S (sigma), coefficient of variation

**Table 3:** Sex- and age-specific percentiles for fat mass index among Chinese adults aged 20–90 years

Age (years)	Males									Females								
			M									M						
	L	S	3rd	10th	25th	50th	75th	90th	97th	L	S	3rd	10th	25th	50th	75th	90th	97th
20	0.748	0.553	0.50	1.37	2.43	3.76	5.22	6.63	8.11	0.641	0.300	2.85	3.70	4.63	5.75	6.96	8.11	9.31
25	0.748	0.537	0.60	1.51	2.61	3.98	5.48	6.93	8.44	0.641	0.301	2.96	3.84	4.81	5.98	7.23	8.43	9.68
30	0.748	0.522	0.71	1.66	2.79	4.20	5.74	7.22	8.76	0.641	0.301	3.07	3.98	4.99	6.20	7.51	8.76	10.05
35	0.748	0.507	0.83	1.81	2.97	4.41	5.98	7.49	9.06	0.641	0.301	3.18	4.13	5.18	6.44	7.79	9.09	10.44
40	0.748	0.492	0.96	1.97	3.15	4.62	6.21	7.74	9.33	0.641	0.302	3.30	4.28	5.37	6.67	8.08	9.43	10.83
45	0.748	0.477	1.09	2.12	3.33	4.80	6.41	7.95	9.54	0.641	0.302	3.41	4.43	5.55	6.91	8.37	9.76	11.21
50	0.748	0.462	1.22	2.27	3.48	4.96	6.56	8.09	9.68	0.641	0.303	3.51	4.56	5.73	7.13	8.63	10.07	11.57
55	0.748	0.447	1.35	2.41	3.61	5.09	6.68	8.19	9.77	0.641	0.303	3.60	4.68	5.87	7.31	8.86	10.34	11.89
60	0.748	0.432	1.48	2.54	3.74	5.19	6.76	8.26	9.80	0.641	0.304	3.67	4.77	5.99	7.46	9.05	10.56	12.14
65	0.748	0.418	1.62	2.67	3.85	5.29	6.83	8.29	9.81	0.641	0.304	3.72	4.84	6.08	7.58	9.19	10.73	12.33
70	0.748	0.403	1.75	2.79	3.96	5.36	6.87	8.31	9.79	0.641	0.305	3.75	4.89	6.15	7.66	9.29	10.85	12.48
75	0.748	0.389	1.88	2.91	4.06	5.44	6.91	8.31	9.75	0.641	0.305	3.77	4.92	6.19	7.72	9.37	10.95	12.60
80	0.748	0.375	2.02	3.04	4.16	5.51	6.94	8.30	9.70	0.641	0.306	3.79	4.95	6.23	7.78	9.44	11.03	12.69
85	0.748	0.361	2.17	3.16	4.26	5.58	6.97	8.29	9.65	0.641	0.307	3.81	4.97	6.27	7.82	9.50	11.11	12.78
90	0.748	0.347	2.31	3.29	4.37	5.65	7.00	8.28	9.60	0.641	0.307	3.82	5.00	6.30	7.87	9.56	11.18	12.86

L (lambda), skewness; M (mu), median; S (sigma), coefficient of variation

**Table 4:** Sex- and age-specific percentiles for appendicular lean mass index among Chinese adults aged 20–90 years

Age (years)	Males									Females								
			M									M						
	L	S	3rd	10th	25th	50th	75th	90th	97th	L	S	3rd	10th	25th	50th	75th	90th	97th
20	1.241	0.110	5.94	6.46	6.98	7.54	8.10	8.59	9.07	−0.220	0.105	4.49	4.77	5.08	5.45	5.85	6.25	6.67
25	1.261	0.111	5.92	6.45	6.97	7.54	8.10	8.60	9.08	−0.115	0.107	4.54	4.84	5.16	5.54	5.96	6.36	6.79
30	1.281	0.112	5.90	6.44	6.97	7.54	8.10	8.60	9.09	−0.010	0.109	4.59	4.89	5.23	5.63	6.05	6.47	6.90
35	1.301	0.113	5.88	6.42	6.96	7.54	8.10	8.60	9.09	0.095	0.110	4.62	4.95	5.29	5.70	6.14	6.56	7.01
40	1.320	0.114	5.85	6.40	6.94	7.52	8.09	8.60	9.08	0.200	0.112	4.65	4.99	5.34	5.77	6.22	6.64	7.09
45	1.339	0.114	5.81	6.37	6.91	7.50	8.07	8.57	9.06	0.306	0.113	4.66	5.01	5.38	5.81	6.26	6.69	7.14
50	1.357	0.115	5.76	6.31	6.86	7.45	8.02	8.52	9.01	0.412	0.114	4.65	5.01	5.38	5.82	6.28	6.71	7.15
55	1.374	0.116	5.69	6.24	6.79	7.37	7.94	8.44	8.93	0.519	0.115	4.63	5.00	5.38	5.82	6.28	6.71	7.15
60	1.389	0.117	5.60	6.15	6.69	7.28	7.85	8.34	8.82	0.627	0.116	4.60	4.97	5.36	5.81	6.27	6.69	7.12
65	1.401	0.118	5.49	6.04	6.58	7.16	7.72	8.22	8.69	0.734	0.116	4.54	4.92	5.32	5.77	6.22	6.64	7.06
70	1.411	0.119	5.37	5.92	6.46	7.03	7.58	8.07	8.54	0.842	0.117	4.47	4.86	5.25	5.70	6.15	6.56	6.97
75	1.419	0.120	5.25	5.79	6.32	6.88	7.43	7.91	8.37	0.950	0.117	4.39	4.78	5.17	5.62	6.06	6.46	6.86
80	1.427	0.121	5.12	5.65	6.17	6.73	7.27	7.74	8.19	1.057	0.117	4.30	4.69	5.09	5.52	5.95	6.34	6.73
85	1.434	0.122	4.98	5.51	6.03	6.58	7.11	7.57	8.01	1.165	0.116	4.22	4.61	5.00	5.43	5.85	6.22	6.59
90	1.441	0.122	4.85	5.38	5.88	6.42	6.94	7.40	7.84	1.270	0.116	4.13	4.52	4.91	5.33	5.74	6.11	6.46

L (lambda), skewness; M (mu), median; S (sigma), coefficient of variation

**Table 5:** Sex- and age-specific percentiles for % BF among Chinese adults aged 20–90 years

Age (years)	Males									Females								
			M									M						
	L	S	3rd	10th	25th	50th	75th	90th	97th	L	S	3rd	10th	25th	50th	75th	90th	97th
20	0.491	0.449	5.58	8.45	11.97	16.61	22.03	27.58	33.68	1.072	0.203	18.16	21.86	25.57	29.64	33.68	37.28	40.81
25	0.589	0.432	5.78	8.93	12.68	17.47	22.86	28.21	33.94	1.136	0.200	18.39	22.20	25.98	30.09	34.12	37.70	41.18
30	0.688	0.415	5.98	9.45	13.43	18.33	23.68	28.84	34.24	1.200	0.198	18.63	22.56	26.40	30.54	34.58	38.13	41.57
35	0.786	0.398	6.19	9.99	14.18	19.18	24.48	29.47	34.58	1.265	0.196	18.88	22.93	26.85	31.03	35.07	38.60	42.00
40	0.884	0.382	6.41	10.55	14.95	20.01	25.24	30.05	34.89	1.329	0.194	19.15	23.33	27.33	31.55	35.60	39.11	42.48
45	0.983	0.365	6.65	11.12	15.69	20.79	25.91	30.54	35.12	1.393	0.192	19.44	23.76	27.83	32.10	36.16	39.66	43.00
50	1.081	0.348	6.91	11.70	16.40	21.50	26.50	30.93	35.26	1.457	0.190	19.72	24.18	28.33	32.65	36.71	40.20	43.52
55	1.179	0.331	7.21	12.31	17.10	22.15	27.01	31.24	35.33	1.521	0.188	19.97	24.56	28.79	33.14	37.21	40.69	43.97
60	1.278	0.314	7.59	12.95	17.80	22.78	27.47	31.52	35.37	1.585	0.186	20.18	24.90	29.20	33.58	37.64	41.09	44.34
65	1.376	0.297	8.07	13.64	18.51	23.39	27.92	31.77	35.40	1.649	0.184	20.35	25.20	29.55	33.95	38.01	41.43	44.63
70	1.475	0.280	8.68	14.40	19.24	24.00	28.36	32.02	35.45	1.714	0.182	20.49	25.46	29.87	34.29	38.32	41.71	44.88
75	1.573	0.263	9.44	15.22	19.99	24.62	28.79	32.27	35.50	1.779	0.180	20.60	25.71	30.17	34.59	38.61	41.97	45.09
80	1.671	0.246	10.36	16.12	20.78	25.25	29.23	32.53	35.58	1.844	0.178	20.71	25.94	30.45	34.88	38.88	42.21	45.28
85	1.770	0.229	11.45	17.09	21.60	25.88	29.67	32.79	35.66	1.909	0.176	20.81	26.17	30.73	35.17	39.15	42.43	45.47
90	1.866	0.213	12.69	18.10	22.43	26.51	30.10	33.04	35.74	1.973	0.174	20.91	26.40	31.00	35.45	39.40	42.66	45.65

*% BF* percentage of body fat mass; L (lambda), skewness; M (mu), median; S (sigma), coefficient of variation

**Fig. 2:**
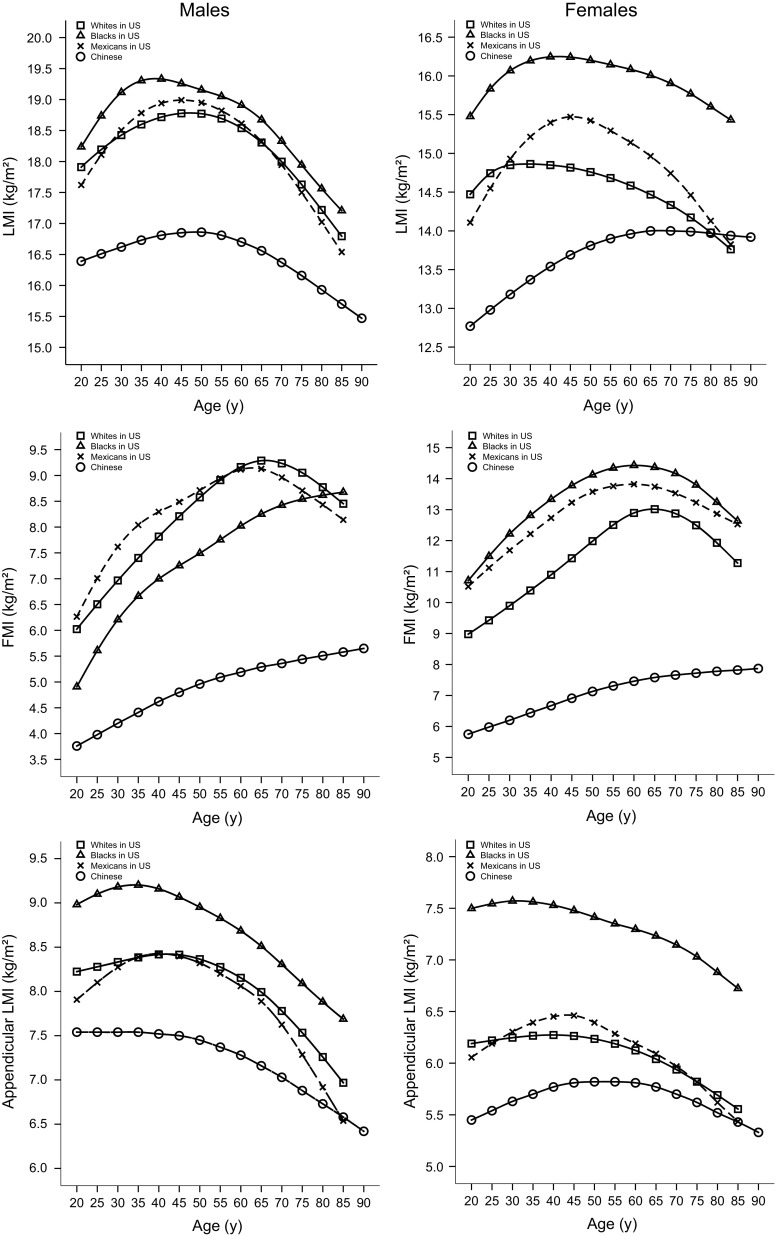
Comparisons of the 50th percentile curves for LMI, FMI, and appendicular LMI according to age and gender for Chinese versus American adults (including white, black, and Mexican adults) from NHANES data. (*LMI* lean mass index, *FMI* fat mass index)

Comparisons with the ethnic groups (including whites, blacks, and Mexicans) of US counterparts from NHANES reference data for the 50th percentile curves of LMI, FMI, and aLMI using the GE-Lunar DXA scanner are shown in Fig. 2. Compared with the white, black, and Mexican populations in the USA, the 50th percentiles of LMI, FMI, and aLMI were consistently distinctly lower in Chinese males and females, with the exception of similar values in older Mexican and white populations for aLMI and LMI. Moreover, the 50th percentiles of aLMI and LMI showed relatively greater decreases in older white and Mexican individuals than in Chinese individuals.

### Percentiles for body fat distribution indices

**Fig. 3:**
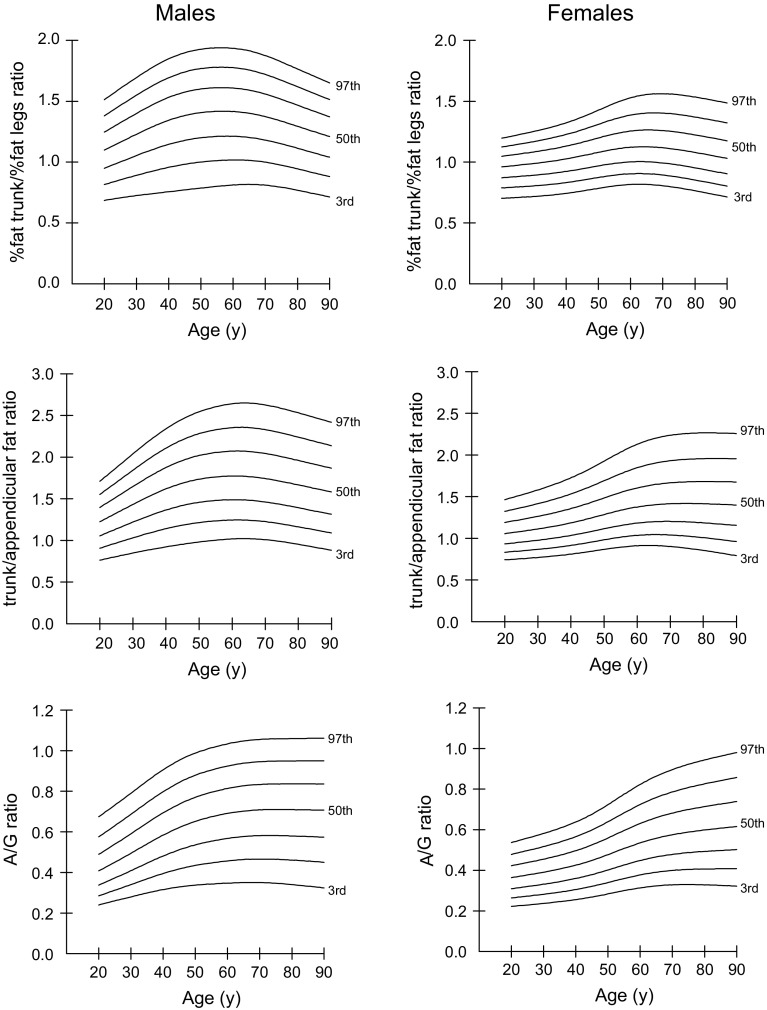
Percentile curves (the 3rd, 10th, 25th, 50th, 75th, 90th, and 97th) for % fat trunk/% fat legs ratio, trunk/appendicular FMR, and A/G FMR in Chinese males and females aged 20–90 years. (*FMR* fat mass ratio, *A/G* android/gynoid)

Percentiles (3rd, 10th, 25th, 50th, 75th, 90th, and 97th) for body fat distribution indices (including % fat trunk/% fat legs ratio, trunk/appendicular FMR, and A/G FMR) for males and females aged 20–90 years are shown in Tables 6, 7 and 8, and percentile curves are given in Fig. 3. The values of % fat trunk/% fat legs ratio, trunk/appendicular FMR, and A/G FMR were consistently higher for males than females. The pattern of changes in the % fat trunk/% fat legs ratio, trunk/appendicular FMR, and A/G FMR differed by sex. The % fat trunk/% fat legs ratio, trunk/appendicular FMR, and A/G FMR increased gradually until the fifth decade in both sexes. These measures decreased thereafter in males, but remained steady in females.

**Table 6:** Sex- and age-specific percentiles for % fat trunk/% fat legs ratio among Chinese adults aged 20–90 years

Age (years)	Males									Females								
			M									M						
	L	S	3rd	10th	25th	50th	75th	90th	97th	L	S	3rd	10th	25th	50th	75th	90th	97th
20	0.989	0.200	0.68	0.82	0.95	1.10	1.25	1.38	1.51	1.341	0.136	0.70	0.79	0.87	0.96	1.05	1.12	1.20
25	1.094	0.204	0.71	0.85	1.00	1.16	1.32	1.46	1.60	1.232	0.140	0.71	0.80	0.88	0.97	1.06	1.14	1.22
30	1.195	0.208	0.72	0.89	1.05	1.23	1.40	1.55	1.69	1.120	0.143	0.72	0.80	0.89	0.99	1.08	1.17	1.25
35	1.286	0.211	0.74	0.93	1.10	1.29	1.47	1.63	1.78	0.999	0.147	0.73	0.82	0.91	1.01	1.11	1.19	1.28
40	1.355	0.212	0.76	0.96	1.15	1.34	1.53	1.69	1.85	0.857	0.150	0.74	0.83	0.92	1.03	1.13	1.23	1.32
45	1.397	0.212	0.77	0.98	1.18	1.38	1.57	1.74	1.90	0.684	0.154	0.76	0.85	0.94	1.05	1.16	1.27	1.37
50	1.410	0.211	0.79	1.00	1.20	1.41	1.60	1.77	1.93	0.481	0.158	0.78	0.87	0.97	1.08	1.20	1.31	1.42
55	1.397	0.209	0.80	1.01	1.21	1.42	1.61	1.78	1.94	0.272	0.162	0.80	0.89	0.99	1.10	1.23	1.35	1.48
60	1.362	0.207	0.81	1.02	1.21	1.41	1.61	1.77	1.93	0.093	0.167	0.82	0.90	1.00	1.12	1.25	1.38	1.53
65	1.321	0.205	0.82	1.01	1.20	1.40	1.59	1.76	1.91	−0.023	0.171	0.82	0.90	1.00	1.12	1.26	1.40	1.55
70	1.290	0.204	0.81	1.00	1.18	1.37	1.56	1.72	1.87	−0.074	0.176	0.81	0.89	0.99	1.12	1.26	1.40	1.56
75	1.276	0.202	0.79	0.97	1.15	1.34	1.51	1.67	1.82	−0.076	0.180	0.79	0.88	0.98	1.10	1.24	1.39	1.55
80	1.276	0.202	0.77	0.94	1.11	1.29	1.47	1.62	1.76	−0.047	0.185	0.76	0.85	0.95	1.08	1.22	1.37	1.53
85	1.280	0.203	0.74	0.91	1.08	1.25	1.42	1.56	1.71	−0.004	0.190	0.74	0.83	0.93	1.06	1.20	1.35	1.51
90	1.285	0.203	0.71	0.88	1.04	1.21	1.37	1.51	1.65	0.043	0.195	0.71	0.80	0.91	1.03	1.18	1.32	1.49

L (lambda), skewness; M (mu), median; S (sigma), coefficient of variation

**Table 7:** Sex- and age-specific percentiles for trunk/appendicular fat mass ratio among Chinese adults aged 20–90 years

Age (years)	Males									Females								
			M									M						
	L	S	3rd	10th	25th	50th	75th	90th	97th	L	S	3rd	10th	25th	50th	75th	90th	97th
20	0.869	0.206	0.76	0.91	1.06	1.22	1.40	1.55	1.71	0.237	0.181	0.74	0.83	0.93	1.06	1.19	1.32	1.46
25	0.888	0.214	0.81	0.97	1.14	1.33	1.52	1.70	1.87	0.184	0.186	0.76	0.85	0.95	1.08	1.23	1.37	1.52
30	0.907	0.221	0.85	1.03	1.22	1.43	1.65	1.84	2.04	0.132	0.191	0.77	0.87	0.98	1.11	1.26	1.42	1.58
35	0.920	0.228	0.89	1.09	1.30	1.53	1.77	1.98	2.20	0.083	0.196	0.79	0.89	1.00	1.15	1.31	1.47	1.65
40	0.919	0.233	0.92	1.14	1.37	1.62	1.88	2.11	2.34	0.034	0.201	0.81	0.92	1.04	1.19	1.36	1.53	1.73
45	0.894	0.237	0.95	1.18	1.42	1.69	1.96	2.21	2.46	−0.018	0.206	0.84	0.95	1.07	1.23	1.42	1.61	1.82
50	0.845	0.240	0.98	1.21	1.46	1.74	2.02	2.28	2.55	−0.072	0.212	0.87	0.98	1.12	1.29	1.48	1.69	1.93
55	0.776	0.243	1.00	1.23	1.48	1.76	2.06	2.33	2.60	−0.114	0.218	0.89	1.01	1.15	1.34	1.55	1.78	2.04
60	0.699	0.244	1.02	1.24	1.49	1.77	2.07	2.35	2.64	−0.126	0.226	0.91	1.04	1.18	1.38	1.60	1.85	2.13
65	0.640	0.246	1.02	1.24	1.48	1.77	2.07	2.35	2.65	−0.099	0.233	0.91	1.04	1.20	1.40	1.64	1.90	2.20
70	0.605	0.247	1.01	1.23	1.46	1.74	2.04	2.33	2.63	−0.035	0.242	0.90	1.04	1.20	1.41	1.67	1.93	2.24
75	0.592	0.250	0.99	1.20	1.43	1.71	2.00	2.29	2.58	0.047	0.250	0.88	1.03	1.20	1.42	1.68	1.95	2.26
80	0.598	0.252	0.95	1.16	1.39	1.67	1.96	2.24	2.53	0.139	0.258	0.86	1.01	1.19	1.41	1.68	1.96	2.26
85	0.615	0.256	0.92	1.13	1.35	1.62	1.91	2.19	2.47	0.238	0.267	0.82	0.99	1.17	1.41	1.68	1.96	2.26
90	0.633	0.259	0.88	1.09	1.31	1.58	1.87	2.14	2.42	0.335	0.276	0.79	0.96	1.16	1.40	1.68	1.95	2.26

L (lambda), skewness; M (mu), median; S (sigma), coefficient of variation

**Table 8:** Sex- and age-specific percentiles for android/gynoid fat mass ratio among Chinese adults aged 20–90 years

Age (years)	Males									Females								
			M									M						
	L	S	3rd	10th	25th	50th	75th	90th	97th	L	S	3rd	10th	25th	50th	75th	90th	97th
20	0.095	0.274	0.24	0.28	0.34	0.41	0.49	0.58	0.67	0.544	0.230	0.22	0.26	0.31	0.36	0.42	0.48	0.54
25	0.235	0.274	0.26	0.31	0.37	0.45	0.54	0.63	0.73	0.529	0.232	0.23	0.27	0.32	0.38	0.44	0.50	0.56
30	0.375	0.273	0.28	0.34	0.41	0.49	0.59	0.69	0.79	0.516	0.234	0.24	0.28	0.33	0.39	0.46	0.52	0.58
35	0.513	0.271	0.30	0.37	0.45	0.54	0.64	0.74	0.85	0.506	0.237	0.25	0.29	0.34	0.41	0.47	0.54	0.61
40	0.645	0.270	0.32	0.39	0.48	0.58	0.69	0.80	0.91	0.501	0.239	0.26	0.31	0.36	0.43	0.50	0.57	0.64
45	0.763	0.268	0.33	0.42	0.51	0.62	0.74	0.84	0.95	0.493	0.241	0.27	0.32	0.38	0.45	0.53	0.60	0.68
50	0.866	0.267	0.34	0.43	0.54	0.65	0.77	0.88	0.99	0.478	0.245	0.28	0.34	0.40	0.48	0.56	0.64	0.72
55	0.948	0.265	0.34	0.45	0.55	0.67	0.79	0.90	1.01	0.462	0.249	0.30	0.36	0.43	0.51	0.60	0.68	0.77
60	1.005	0.264	0.35	0.46	0.57	0.69	0.81	0.92	1.03	0.453	0.253	0.31	0.38	0.45	0.54	0.63	0.72	0.82
65	1.040	0.264	0.35	0.46	0.58	0.70	0.83	0.94	1.05	0.453	0.257	0.32	0.39	0.47	0.56	0.66	0.76	0.86
70	1.061	0.264	0.35	0.47	0.58	0.71	0.83	0.95	1.05	0.467	0.262	0.33	0.40	0.48	0.58	0.68	0.79	0.90
75	1.076	0.265	0.35	0.46	0.58	0.71	0.84	0.95	1.06	0.493	0.268	0.33	0.40	0.49	0.59	0.70	0.81	0.92
80	1.093	0.268	0.34	0.46	0.58	0.71	0.84	0.95	1.06	0.527	0.273	0.33	0.41	0.49	0.60	0.71	0.83	0.94
85	1.114	0.272	0.33	0.46	0.58	0.71	0.84	0.95	1.06	0.565	0.279	0.33	0.41	0.50	0.61	0.73	0.84	0.96
90	1.137	0.275	0.32	0.45	0.57	0.71	0.84	0.95	1.06	0.603	0.285	0.32	0.41	0.50	0.62	0.74	0.86	0.98

L (lambda), skewness; M (mu), median; S (sigma), coefficient of variation

**Fig. 4:**
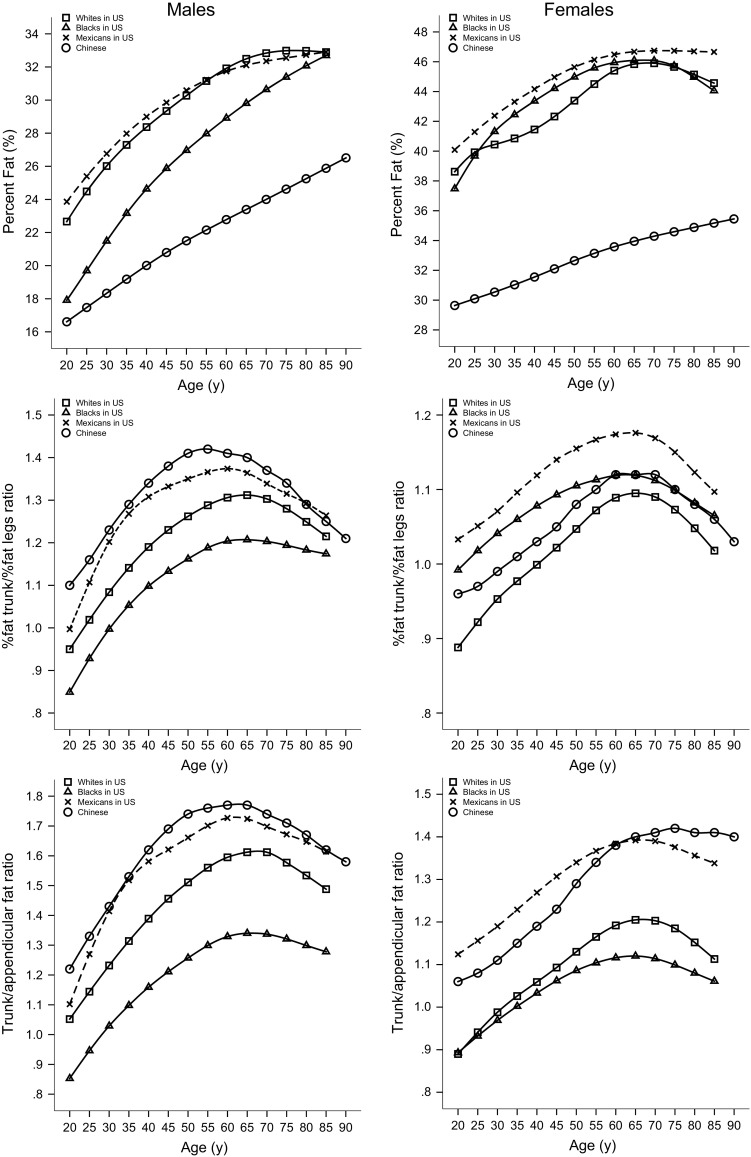
Comparisons of the 50th percentile curves for % body fat, % fat trunk/% fat legs ratio and trunk/appendicular FMR according to age and gender for Chinese versus American adults (including white, black, and Mexican adults) from NHANES data. (*FMR* fat mass ratio)

Figure 4 shows the 50th percentile curves of % BF, % fat trunk/% fat legs ratio and trunk/appendicular FMR using the GE-Lunar DXA scanner for white, black, and Mexican populations in the USA using NHANES reference data. The 50th percentiles of % BF were consistently distinctly lower in Chinese males and females; however, body fat distribution indices for Chinese adults were not parallel with US data in terms of total FM for either sex. Particularly, Chinese men had greater central adiposity (% fat trunk/% fat legs ratio and trunk/appendicular FMR). The 50th percentiles of the % fat trunk/% fat legs ratio and trunk/appendicular FMR were consistently higher in Chinese males than their US counterparts, with the exception of similar values in Mexican individuals aged 80–85 years. The 50th percentiles of % fat trunk/% fat legs ratio were consistently higher in Chinese women than white American women, whereas these measures were lower than those of black and Mexican women, except for black women aged 55–85 years. The 50th percentiles of trunk/appendicular FMR were consistently higher in Chinese women than their American counterparts, with the exception of lower values than Mexican women aged 20–60 years.

## Discussion

The present study provides sex- and age-specific curves and percentiles for LMI, FMI, aLMI, and body fat distribution indices using DXA in Chinese adults. First, to the best of our knowledge, there are no previous studies establishing values for LMI, FMI, aLMI, and body fat distribution indices using DXA based on a large sample of Chinese adults. Using these percentile values provides more accurate assessments of nutritional status (obesity and sarcopenia) for Chinese men and women. Second, in comparison with white, black, and Mexican populations in the USA as reported in the NHANES reference data [12], Chinese adults had lower total FM than their US counterparts, but regional body fat distribution indices were not parallel with US data in terms of total FM for either sex. Particularly, Chinese men had greater central adiposity (% fat trunk/% fat legs ratio and trunk/appendicular FMR). Moreover, older white and Mexican populations had relatively greater decreases for LMI than those of the surveyed Chinese population.

### Values for LMI and FMI by DXA

In the present study, LM and FM were normalized by height^2^, similar to BMI, which is weight divided by height^2^. LMI, especially aLMI, as a measure of abnormally low muscle mass has been widely applied in the diagnosis of sarcopenia [8, 9]. FMI values can be used to assess clinical obesity as proposed by Kelly [11] for identifying subjects with high obesity disease risks and for enrolling high-risk individuals into clinical trials.

Consistent with previous studies [11–13], our data demonstrate sex differences in LM and FM throughout the entire life span, with males having greater LM and lower FM than females. Moreover, the patterns of age-related changes for LM and FM were different in Chinese men and women. In the present study, the LMI and aLMI for Chinese men were negatively correlated with age and an evident decease was observed from the fifth decade. In Chinese women, LMI and aLMI showed no relationship with age. Other studies showed both males and females experience age-related deceases in appendicular LM as measured by DXA [20] and whole-body LM as measured by MRI [21], and that males typically experience greater deceases than females for LM. Previous studies in Asian populations have shown the prevalence of sarcopenia in men was higher than that in women [22, 23]; therefore, males may be more likely to develop sarcopenia than females in Asian populations. Consistent with the NHANES data [11, 12], FMI increased with age in Chinese men and women. However, FMI had no relationship with age in Korean men [13]. Sex differences in LM and FM are distinct early in life, and become much more marked during puberty as shown in our previous study [14]. These sex differences in body composition may be mainly attributed to the action of sex steroid hormones, which drive the dimorphisms during pubertal development [24].

Ethnic differences in body composition have been reported in previous studies [11, 12]. Compared with their American counterparts as reported in the NHANES data, the values of LMI, aLMI, and FMI were distinctly lower in Chinese males and females, with the exception of similar values recorded for older Mexican and white individuals for aLMI and LMI. An interesting finding was that in both sexes, older white and Mexican populations had greater deceases for aLMI and LMI than Chinese. Older Chinese adults today may have performed more physical activities in their lifetimes than younger Chinese populations because they may have experienced more difficult living conditions since early adulthood, thus their muscle mass may be in better condition than that of younger generations. This may have led to the older Chinese having lower deceases for LM than expected.

### Values for body fat distribution indices

Over and above fat mass per se, the pattern of body fat distribution is an independent and stronger predictor of health risk [17, 25]. Previous studies have revealed that android (namely central, upper body or truncal) adipose deposition is related with an increased risk of metabolic and cardiovascular diseases [17, 26], while gynoid (namely gluteal-femoral or lower body) fat tissue is associated with reduced metabolic risk and may be protective against adverse health effects in both sexes [17, 25].

Sex differences in body fat distribution have been well studied. In accordance with previous studies [11–13], we found males accumulated more central adiposity (% fat trunk/% fat legs ratio and trunk/appendicular FMR) than females throughout the entire life span, though females generally have higher total adiposity relative to males. Ethnic differences in fat distribution have been reported previously [11, 12], with white and Mexican populations in the USA having relatively higher central fat than black populations. Previous investigations in Asian populations have demonstrated that Asian ethnicity is associated with higher central adiposity than in Caucasian populations [27, 28], even in childhood [29]. Compared with their American counterparts, we discovered Chinese adults had distinctly lower total FM, while the central fat distribution indices were not parallel with the US data in terms of total FM for either sex. Particularly, Chinese men had more central adiposity. The values and patterns of change in the body fat distribution indices in Chinese adults were similar to those of their Korean counterparts [13].

## Limitations

There are several potential limitations in this study. First, the cross-sectional design does not allow for the further longitudinal assessment of body composition accrual in individuals. Second, individuals with extreme underweight (BMI < 16 kg/m^2^) or obesity (BMI ≥ 30 kg/m^2^) were excluded, and the subjects in this study were not randomly selected, despite the large sample (*n* = 5688). Thus, these data may not be representative of the whole national population. However, we feel that the data were fairly representative of the population in terms of median BMI of both sexes. The median BMI was 22.5 kg/m^2^ for males and 22.1 kg/m^2^ for females in this study, compared with a BMI of 22.8 kg/m^2^ for males and 23.3 kg/m^2^ for females in a randomly selected population of the Working Group on Obesity in China [30]. Additionally, estimates of body composition using different DXA scanners may show substantially different DXA values. Our data are limited only to results derived from the Lunar Prodigy DXA; these percentile curves may be improperly used with results from other brands of DXA scanners. Finally, caution should be advised in using the present values because of the differences in nutritional habits, genetic backgrounds, physical activity levels, and other lifestyle factors of the study population.

In conclusion, we present the sex- and age-specific percentiles for aLMI, LMI, FMI, and body fat distribution indices using DXA in Chinese adults. These body composition indices may refine the individual assessment of the nutritional status of adults, and serve as a useful tool for public health screening with regard to Asian populations. These indices may also allow comparisons of future national and international epidemiological studies, as well as the prevention and recognition of obesity, undernutrition, and sarcopenia.

## Abbreviations

DXA: Dual-energy X-ray absorptiometry
LM: Lean mass
FM: Fat mass
LMI: Lean mass index
FMI: Fat mass index
aLMI: Appendicular lean mass index
FMR: Fat mass ratio
A/G: Android/gynoid
% BF: Percentage of body fat mass
